# Metagenome-Assembled Genome HetDA_MAG_MH13 of the Family *Devosiaceae*, from a Marine N2-fixing Cyanobacterial Enrichment Culture

**DOI:** 10.1128/mra.00452-22

**Published:** 2023-01-26

**Authors:** Harold Carlson, Elaina D. Graham, John F. Heidelberg, Eric A. Webb

**Affiliations:** a Department of Biological Sciences, University of Southern California, Los Angeles, California, USA; Indiana University, Bloomington

## Abstract

Here, we present a draft genome in the order *Rhizobiales* and family *Devosiaceae*. This draft genome comes from an enrichment of a heterocystous, cyanobacterial diazotroph (HetDA) that was originally living in association with *Trichodesmium* species. This *Rhizobiales* organism is proposed to be an anoxygenic phototroph capable of dissimilatory nitrate reduction to ammonia (DNRA).

## ANNOUNCEMENT

Diazotrophic cyanobacteria such as *Trichodesmium* species are primary producers in the oligotrophic ocean (1). *Trichodesmium* species can live in association with a complex community of microorganisms, including a *Calothrix*-like heterocystous diazotroph (HetDA) (2). The ecological role of this community in biogeochemical cycles needs further investigation. To this end, here we present a metagenome-assembled genome (MAG) from an enrichment of a HetDA consortium from the North Pacific Subtropical Gyre.

Samples were collected as detailed by Momper et al. (2). Briefly, *Trichodesmium* colonies were isolated and transferred into sterile YBC II medium at 24°C with a 12-h light (100 μmol photons m^−2^ s^−1^)/12-h dark cycle. The enrichment was grown under the aforementioned conditions for 5 years prior to sequencing and was transferred to fresh medium every month. A 50-mL subsample was taken from the enrichment, and DNA was extracted using a Qiagen DNeasy PowerSoil kit. Extracted DNA was sent to the University of Southern California Epigenome Center and sequenced on an Illumina MiSeq sequencer (paired-end 250-bp reads, with a raw read total of 4,725,335 reads) using the MiSeq reagent kit v2 with 300 cycles. Reads were trimmed using Trimmomatic v0.38 (parameters: –phred33, ILLUMINACLIP:TruSeq3-PE.fa:2:30:10 SLIDINGWINDOW:10:28 MINLEN:50) (3), assembled using MEGAHIT v1.2.8 (parameters: –presets meta-sensitive) (4), and mapped to the assembly using Bowtie2 v2.3.5 (5). Mapped reads were filtered using CoverM v0.4.0 (parameters: –min-read-percent-identity 0.95 –min-read-aligned-percent 0.75) (6). The assembly was binned using MetaBAT2 v2.12.1 (7), BinSanity v0.3.8 (8), and Concoct v1.1.0 (9), with default parameters for each, and DASTool v1.1.2 (10) was used to determine a final set of MAGs. All bins were run through MetaSanity v1.3.0 and FuncSanity (11) to generate functional annotations and PhyloSanity (11) to determine novelty based on GTDB-Tk v1.1.0 (r89) relative evolutionary divergence (RED) scores (12). HetDA_MAG_MH13 was assigned to the family *Devosiaceae* by GTDB-Tk. It is 96.88% complete and 3.159117 Mbp long, with a predicted complete length of 3.260856 Mbp, as determined by CheckM (13). The MAG consists of 319 scaffolds, with an $N_{50}$ value of 14,691 bp, a GC content of 63.3%, 3,248 predicted genes, and a coding density of 91.01%.

HetDA_MAG_MH13 is suggested to be a photoorganoheterotroph. It possesses the *pufM* and *pufL* genes (KEGG orthology numbers K08929 and K08928, respectively), which correspond to the anoxygenic type II reaction center, suggesting that it has the potential for anoxygenic phototrophy (14). No evidence of the Calvin cycle, hydrogenases, or sulfide oxidation capabilities was detected, suggesting that HetDA_magMH13 may be a heterotrophic aerobic anoxygenic phototroph (15).

HetDA_MAG_MH13 also has the genes for dissimilatory nitrate reduction to ammonia (DNRA), without hydrogenases and sulfide-oxidizing capabilities. These data suggest, consistent with previous DNRA research (16), that the MAG oxidizes carbon in DNRA. Since DNRA requires anoxic conditions, this indicates that the HetDA consortium may transiently contain an anoxic microenvironment. This finding is consistent with previous research on chemical gradients in *Trichodesmium* consortia (17, 18). DNRA is more thermodynamically favorable than denitrification under nitrate-limited anoxic conditions. Marine DNRA is an understudied part of the nitrogen cycle, and its presence in an oligotrophic ocean isolate MAG suggests that its role extends beyond oxygen minimum zones and sediments.

In summary, HetDA_MAG_MH13 appears to be in the family *Devosiaceae*, with the potential for anoxygenic phototrophy and DNRA.

### Data availability.

Raw sequences and MAGs are available under BioProject accession number PRJNA719568. Raw reads are available under SRA accession number SRR14140256, and MAGs are available under BioSample accession number SAMN18613311.
